# Early malperfusion, ischemia reperfusion injury, and respiratory failure in acute complicated type B aortic dissection after thoracic endovascular repair

**DOI:** 10.1186/1749-8090-8-17

**Published:** 2013-01-23

**Authors:** Jiang Xiong, Minhong Zhang, Wei Guo, Xiaoping Liu, Tai Yin, Xin Jia, Hongpeng Zhang, Yongle Xu, Lijun Wang

**Affiliations:** 1 Departments of Vascular Surgery, Clinical Division of Surgery, Chinese PLA General Hospital, Beijing, China

## Abstract

**Background:**

The aim of this study was to determine the early mortality and major complications of acute complicated type B aortic dissection (ACBD) after thoracic endovascular aortic repair (TEVAR).

**Methods:**

Twenty-six consecutive patients with ACBD who underwent TEVAR were included. Clinical indications before TEVAR and in-hospital mortality and major complications after TEVAR were analyzed and compared with similar reports.

**Results:**

TEVAR was technically successful in all cases. In-hospital mortality occurred in four patients (15%), and major complications occurred in an additional four patients (15%). Three of the four (75%) of the deaths were associated with malperfusion and ischemia reperfusion injury (IRI), and 3/4 (75%) of the major complications were caused by respiratory failure (RF).

**Conclusions:**

In-hospital mortality associated strongly with severe end-organ malperfusion and IRI, while major complications associated with RF, during TEVAR. Our results indicate that malperfusion, IRI and respiratory failure during TEVAR should be carefully monitored and aggressively treated.

## Background

Thoracic endovascular aortic repair (TEVAR) is the promising treatment for acute complicated type B aortic dissection (ACBD) [1-4]. During the acute phase of the dissection, TEVAR can obviate impending aortic rupture and relieve dynamic malperfusion [5,6]. A delayed benefit is potentiation of thrombosis within the thoracic false lumen, thereby mitigating the risk of aneurysmal dilatation and subsequent aortic rupture [7-9]. TEVAR is superior to conventional surgical repair, which is more invasive and has more potential complications.

Despite the short-term benefits (low morbidity and mortality) of TEVAR for the repair of ACBD [1,4], an increasing number of studies report early mortality and major complications after TEVAR [7,10-12]. Major complications of TEVAR include permanent renal failure, stroke, paraplegia, and respiratory failure (RF). An analysis of early mortality and major complications, with the exclusion of procedure-related factors, would provide a better understanding of the impact of TEVAR on ACBD outcomes and provide insight in the clinical decisions of when to use TEVAR and when to add adjuvant therapy to the TEVAR procedure. Therefore, this study focused on ACBD patients to identify indicators that could predict in-hospital mortality or major complications during TEVAR.

## Methods

### Patients

From March 2004 to October 2010, 26 consecutive patients with ACBD underwent TEVAR in our department. TEVAR was performed less than 14 days after the onset of symptoms in all cases. The clinical characteristics of the patients are listed in Table 1. All the patients had confirmed Stanford type B dissection by computed tomography (CT) angiography. ACBD was diagnosed according to the signs and symptoms, including severe end-organ malperfusion, persistent pain, acute aortic failure, refractory hypertension and encephalopathy.

**Table 1:** Baseline characteristics and CT findings

**Variable**	**Number (%)**
Sex (Male/ Female)	23/ 3
Age (y; Mean ± SD (Range))	52.8 ± 13.1 (31–72)
Descending aorta dimension (mm; Mean ± SD (Range))	29.0 ± 2.7 (23–36)
Patients in distance from LSCA to primary tear	
< 1.5 cm	13 (50.0)
> 1.5 cm	13 (50.0)
True lumen appearance	
Patent	20 (76.9)
Severe Stenosis (location) ^#^	2 (7.7)
Thoracic aorta	1 (3.8)
Common iliac	1 (3.8)
Occlusion	4 (15.4)
Abdominal aorta below the renal artery	1 (3.8)
Common iliac	3 (11.5)

LSCA- left subclavian artery; ^#^ Diameter decreased more than 80%.

### Techniques

Before the endovascular procedure, CT angiography was used to measure the distance and diameter of the landing zone, which is the normal part of the aorta used to attach the stent graft during the TEVAR procedure. During the procedure, the landing zone diameter was determined by digital subtraction angiography. The diameter of the selected stent graft was 10-20% larger than the landing zone diameter. All procedures were performed in an angiography unit (INNOVA 3100, GE Medical Systems, Milwaukee, WI). Local anesthesia was used in two cases due to pre-existing cardiac and respiratory dysfunction, while general anesthesia was performed in the remaining 24 cases.

All stent grafts were deployed with the common femoral artery approach via unilateral femoral access. It was not necessary to use conduits for any of the cases. The technical details of TEVAR have been described by Nienaber [13]. Once the true lumen wire access through the left brachial artery was confirmed in the ascending aorta, arch angiography was performed. If imaging indicated the need for left subclavian artery coverage, selective vertebral angiography was performed to evaluate the adequacy of collateral vertebral artery flow and to confirm the absence of internal mammary grafts.

Patients were systemically heparinized with a dosage of 100 IU/kg by intravenous bolus injection. The delivery system dilator–sheath was advanced into the aortic true lumen, and the stent graft was advanced to completely cover the primary intimal tear. The systolic blood pressure was lowered to less than 100 mmHg, and the heart rate was lowered to below 90 beats/min. In order to exclude false lumen flow, the stent graft was delivered under fluoroscopic guidance to cover the primary intimal tear. Ballooning of the stent graft was performed only if a large type Ia proximal endoleak was documented, and ballooning occurred only at the proximal landing zone. When needed, a second stent graft was placed. Angiography confirmed the absence of endoleaks and determined the perfusion status of the previously ischemic arterial beds. Technical success was determined by exclusion of proximal primary entry flap on the completion angiogram. The details of TEVAR procedures are listed in Table 2.

**Table 2:** TEVAR procedure characteristics

**Variable**	**Number (%)**
Time interval between symptom onset and procedure	
(d; Mean ± SD (Range))	7.8 ± 4.4 (1–14)
Stent graft oversizing aorta diameter of landing zone	
10%	20 (76.9)
15%	4 (15.4)
20%	2 (7.7)
Size of the stent graft ^#^ (mm; mean ± SD (Range))	
Proximal diameter	33.7 ± 3.6 (28–40)
Distal diameter	33.4 ± 3.8 (28–40)
Graft length	127.6 ± 29.2 (60–162)
Coverage of branch artery	
LSCA coverage	9 (34.6)
LSCA dominant	2 (7.7)
Aberrant RSCA coverage	1 (3.8)
LCCA coverage	2 (7.7)
Partial LCCA coverage	1 (3.8)
Length of aorta covered (cm; mean ± SD (Range))	13.5 ± 2.9 (6–18)
Operation time (h; mean ± SD (Range))	2.2 ± 1.1 (1–6.4)
Contrast volume (ml; mean ± SD (Range))	190 ± 61.2 (100–350)

LSCA- left subclavian artery; RSCA- right subclavian artery; LCCA- left common carotid artery.

^#^ Two stent grafts were implanted in one patient.

ACBD patients with severe end-organ malperfusion who underwent TEVAR were administered prophylactic hemodialysis. In the case of ACBD with severe malperfusion, the important organs affected were the kidney, the intestine and the lower extremities. Severe end-organ malperfusion was empirically defined as the appearance of any one of following indicators: Type II extremity ischemia [14], intestine malperfusion related hematochezia or hematemesis, blood creatinine level ≥ 500 μmol/L, or blood creatine kinase ≥ 10000 IU/L. According to empirical indicators of the severe end-organ malperfusion, patients with more than two indicators were administered prophylactic hemodialysis after TEVAR.

### Data collection and statistical analysis

Data collected included age, survival status, graft components utilized, access arteries, subclavian artery coverage, length of in-hospital stay, major/minor in-hospital complications, and status of the false lumen immediately after procedure. Major complications were defined as events that were life threatening or would prompt major therapeutic consequences (e.g., access complications requiring surgical revision, re-interventional treatment or requiring dialysis). Minor complications were defined as that no further treatment was required (e.g., transient renal failure not requiring dialysis or transient spinal cord ischemia).

This was a retrospective, non-comparative analysis. All statistical data were descriptive on an intent-to-treat basis. Discrete variables were presented as percentages, while continuous variables were presented as counts and are presented as mean ± SD. The ethical approval for this study was provided by the Ethical Committee of the Chinese PLA General Hospital.

## Results

### Procedural data

Symptomatic indications for intervention included persistent pain (23 patients), malperfusion (10 patients), aortic failure (8 patients), and refractory hypertension (5 patients). Multiple indicators were present in 16 patients (Table 3).

**Table 3:** Indicators for TEVAR

**Indications**	**n**
Malperfusion	10 (38.5%)
Lower extremity ischemia	4
Paraplegia	2
Renal artery malperfusion	7
SMA malperfusion	6
Refractory hypertension/ encephalopathy	5/2 (19.2%)
Persistent Pain	23 (88.5%)
Chest pain	14
Abdominal pain	16
Aortic failure	8 (30.8%)
Acute true lumen collapse	3
Severe pleural effusion	6

Twelve patients had two indications and four patients had three indications.

SMA: superior mesenteric artery.

Six types of stent graft systems were used, including Talent (Medtronic Inc, Minneapolis, MN; n = 11), Valiant (Medtronic Inc, Minneapolis, MN; n = 8), Zenith (COOK, Inc, Bloomington, IN; n = 1), Endofit (Endologix Inc, Irvine, CA; n = 3), Hercules (Microport, Shanghai, China; n = 4) and Ankura (Lifetech, Shenzhen, China; n = 2). One stent graft was implanted in 25 patients, and two stent grafts were implanted in one patient to cover multiple tears on the distal descending aorta. The coverage distance between the left common carotiod artery (LCCA) and the primary tear was less than 15 mm in two patients.

Technical success was achieved in all patients. Delivery system via the femoral approach was successful in all 26 patients, requiring unilateral incision in 24 cases, bilateral femoral incisions in one case, and unilateral incision with contralateral percutaneous access in one case. Immediate postoperative angiography showed no re-entry site at the distal end of the false lumen of six patients with ACBD. The blood supply of visceral arteries via the false lumen was found in the remaining 20 cases.

Twenty patients (77%) required intensive care unit (ICU) monitoring and continuous intravenous infusion of antihypertensive agents. The average length of hospital stay was 13.8 ± 9.9 days (range, 1 to 38 days).

Based on the angiographic findings and patient symptoms, all patients with malperfusion had immediate resolution of the malperfusion deficit after the procedure. Endoleaks were detected in six patients using procedural angiography; five with Type Ia endoleak, in which left subclavian artery was covered in three cases, and one with a Type II endoleak. All patients with endoleaks were untreated in the procedure.

### In-hospital deaths and major complications

Clinical data for the patients who died or had major complications are summarized in Table 4. Four patients died and four patients experienced major complications following TEVAR. In these patients, the average length of stay in the ICU was 12.9 ± 9.8 days (range, 0 to 29 days) after the procedure. Three deaths were attributed to pre-existing organ malperfusion and ischemia reperfusion injury (IRI), and one was attributed to RF, respiratory infection, and renal failure (Table 4). An independent Clinical Events Committee adjudicated that all deaths were not related to the TEVAR procedure itself.

**Table 4:** Patient outcomes

**Outcome**	**Sex/ Age**	**Clinical indications**	**Indicators of severe end-organ malperfusion (number)**	**Cause of death or major complication**	**Malperfused branch arteries before procedure**	**Onset time to procedure**	**Malperfused branch arteries after procedure**	**Length of stay post procedure**
Death	M/ 58	Abdominal pain, paraplegia, bilateral low extremity ischemic mottling (type III) ^a^, arterial hypertension	3	IRI, MOF	Occlusion of bilateral RA and infrarenal aorta true lumen collapse	48 h	Open of left RA (double lumen supply), right RA (false lumen supply) and infrarenal aorta (true lumen supply)	9 d (ICU 9d)
Death	M/ 39	Abdominal pain, hematochezia, hematemesis	3	IRI, MOF	Occlusion of SMA, bilateral RA	15 h	Open of bilateral RA (true lumen supply), SMA dissection	2 d (ICU 2d)
Death	M/ 31	Abdominal pain, hematochezia, left low extremity ischemic pallor and suggillation (type III), arterial hypertension	3	IRI, Intestinal tract necrosis	Occlusion of SMA, right RA and right CIA	13 h	Open of SMA,right RA and right CIA (true lumen supply)	7 h (ICU 7 h)
Death	M/ 61	Arterial hypertension, encephalopathy	no	ARDS, renal failure, respiratory infection, encephalopathy	None	8 d	None	29 d (ICU 29 d)
Live	M/ 65	Chest pain, arterial hypertension	no	ARDS, acute hepatic failure, respiratory infection	None	52 h	None	31 d (ICU 24d)
Live	M/ 41	Tar stool	2	ARDS, respiratory infection, hydropericardium, pleural effusion	Occlusion of right RA and CIA	11 d	Open of right RA and CIA (true lumen supply)	12 d (ICU 11d)
Live	M/ 32	Obesity, pleural effusion	no	ARDS, Respiratory infection	None	7 d	None	34 d (ICU 16d)
Live	F/ 39	Abdominal pain, hematochezia	2	Intestinal ischemia, intestinal infection, ischemic pancreatitis	Occlusion of SMA, right RA and left EIA	4 d	Open of SMA, right RA and left EIA (true lumen supply)	38 d (ICU 11d)

RA, renal artery; SMA, superior mesenteric artery; CIA, common iliac artery; MOF, multiple organ failure; ARDS, adult respiratory distress syndrome; EIA, external iliac artery; IRI, ischemic reperfusion injury. ^a^The classification of low extremity ischemia was from reference 14.

In six patients with SMA malperfusion and a bloody stool, two died and one had a major complication after the procedure. Of the other three patients, all survived without any symptoms of intestinal ischemia. In four patients with lower extremity malperfusion, two patients had Type III ischemic mottling who died after the procedure. One patient had no pulse, pallor, and paresthesia, but he survived once the lower extremity malperfusion was relieved. In seven patients with renal malperfusion before the procedure, only one patient had renal dysfunction after the procedure. Three patients with three indicators of severe end-organ malperfusion died even though hemodialysis was administered after TEVAR (Table 4).

In four patients with major complications after the procedure, three had RF and respiratory infection. Another patient had pre-existing organ malperfusion and IRI. In four patients with RF after the procedure, the duration of tracheal intubation was 20 ± 7.8 days (range, 11 to 29). Only one patient had severe pleural effusion before the procedure. In six patients with severe pleural effusion after the procedure, five accepted tracheal extubation immediately after the procedure, while one patient with tracheal intubation for 16 days had RF and respiratory infection.

Two patients had minor complications. One patient had a headache that resolved five days after the procedure. The other patient had right low extremity malperfusion and transient renal failure, but recovered one week after the procedure without the need for dialysis. In 23 patients with persistent chest or abdominal pain before the procedure, three died from malperfusion and IRI, while three of the survivors had persistent chest and abdominal pain that was unrelieved. In five patients with refractory hypertension before the procedure, one died. Of the remaining four survivors, three patients had refractory hypertension that resolved on its own. Except for one pre-procedural paraplegia patient, none of the surviving patients had complications requiring further treatment at the time of hospital discharge.

### Follow-up data

All 22 in-hospital live patients were followed up from 1 to 49 months (average, 6.8 ± 7.3 months). The type Ia endoleak in 4 patients spontaneous resolved at one month. The endoleaks in another 2 patients (1 with type Ia and 1 with type II) were observed at three month. Two deaths occurred at 2 and 3 months and the reasons were unclear. One late deaths was as the result of pulmonary carcinoma at 24 months.

## Discussion

Both dissection rupture or imminent rupture and malperfusion are indicators for emergency TEVAR in a patient with ACBD [15]. In fact, imminent rupture is difficult to determine clinically in a patient with ACBD, and dissection rupture is more rare than malperfusion [9,15,16]. The most significant findings of this study were that in-hospital mortality associated with severe end-organ malperfusion IRI and major complications associated with RF during TEVAR.

There were 10 patients (38.5%) with malperfusion in our group; however, no patients showed evidence of dissection rupture or imminent rupture. In previous single-center studies, malperfusion was a common indicator among patients (Table 5). Before our study, the poor prognosis of malperfusion had not been systematically analyzed. Compared with other indicators, including refractory hypertension, persistent pain and aorta failure, malperfusion is a more urgent indicator for emergency TEVAR. This is due to the fact that end-organ malperfusion and subsequent life-threatening organ failure are imminent results. In addition, the severity of ACBD malperfusion and IRI appear to be more closely related to early mortality following TEVAR than the other complications.

**Table 5:** Malperfusion proportion in ACBD patients

**Study**	**Malperfusion (n)**	**ACBD (n)**	**Proportion**
Szeto [4]	17	35	48.6
Feezor [6]	11	33	33.3
Conrad [7]	17	33	51.5
Parsa [12]	11	22	50
Pearce [15]	8	15	53.3
Khoynezhad [16]	15	28	53.6
Botsios [17]	6	32	18.8

ACBD = acute complicated type B aortic dissection.

After TEVAR, the malperfused end-organ artery may open causing early IRI. Irreversible malperfusion and early IRI can increase the systemic absorption of metabolic toxins, which may become a direct cause of multiple organ failure and death after TEVAR. If end-organ malperfusion is more serious, then the IRI will be more serious after TEVAR and early mortality will consequently be higher. In-hospital mortality following TEVAR occurred in three of the 10 patients with malperfusion and accounted for three of four (75%) in-hospital deaths. In similar single-center studies, Pearce [16] and Szeto [4] reported a higher proportion of malperfusion (Table 5), but no malperfusion-related mortality. In other similar two single-center studies, Khoynezhad [17] and Feezor [6] reported a higher proportion of malperfusion (Table 5) and significant malperfusion-related mortality rates (7% and 13.3%, respectively). In our study, the three malperfusion-related deaths occurred in patients who had severe malperfusion and IRI of one or two end-organs. In studies by Pearce [16], Szetodl [4], Khoynezhad [17] and Feezor [6], information regarding the degree of end-organ ischemia was not included. Classification of the degree of end-organ malperfusion would help predict the mortality rate after TEVAR.

In the process of TEVAR, many surgeons use surgical or interventional fenestration [18,19] or branch vessel stenting [6] to handle persistent malperfusion. There are few reports about the process of IRI in malperfusion with an open end-organ artery.

In our study, three of five ACBD patients with severe malperfusion suffered IRI, multiple organ failure, and in-hospital death even though they received prophylactic hemodialysis post-TEVAR. We found that the clearance of metabolic toxins was poor in improving survival of severe malperfusion ACBDs. For these patients, removal of severe malperfusion end-organs may be acquired more beneficial than re-opening the end-organ artery. Currently, more published reports of ACBD have been concerned how to reopen malperfusion end-organ arteries in TEVAR [3,6,15,20-24]. Fenestration and stenting have been used frequently, and prosthetic bypass has been used in visceral malperfusion.

There are few reports detailing when malperfusion persists to result in end-organ ischemia, nor are there any reports regarding the assessment of the degree of end-organ malperfusion, IRI severity and reversibility of malperfusion end-organ function in TEVAR therapy. In our study, the cause of three ACBD patient deaths were malperfusion and IRI, who had more than two indicators of severe end-organ malperfusion (Table 4). On the other hand, two malperfusion ACBD patients who survived had only two indicators (Table 4). The other five malperufsion ACBD patients who survived had no indicators of severe end-organ malperfusion. Re-opening the malperfusion artery is not a unique option. We propose that nonreversible end-organ malperfusion is an indication for primary surgical removal of end-organs (e.g., extremity amputation, intestine resection). Some centers have reported a little experience of organ resection, but the organ removal indicators, primary surgical risk and postoperative prognosis is still unknown [9,21]. Our study suggests that a number of indicators of severe end-organs malperfusion could become the basis of malperfusion organ removal or malperfusion organ artery re-opening.

RF occurred postoperatively in four patients, including one who died. The cause of postoperative RF was not clear because these patients had no pulmonary disease or severe malperfusion, with the exception of one patient who had preoperative pleural effusion and obesity. In regards to the possible causes of respiratory failure, it has been reported that ischemia and reperfusion of intestine, renal, and lower extremity may cause distant pulmonary injury. Feezor et a.l [6] reported that 11 of 33 ACBD patients who underwent emergency TEVAR suffered RF without any apparent underlying cause. Additionally, Sachs [25] showed respiratory complications accounted for 4.3% of type B dissections after TEVAR. Kurabayashi [26] demonstrated that the only independent predictor of oxygenation impairment was acute aortic dissection (odds ratio, 1.323; 95% confidence interval, 1.035-1.691, P = 0.026). This study proposed that RF in acute aortic dissection closely correlated with aortic injury, possibly mediated by the magnitude of the systemic inflammatory reaction to the aortic injury. Eggebrecht [27] quantified inflammatory markers in patients who underwent TEVAR and found that post-procedural inflammatory responses were more pronounced and correlated with mortality. Furthermore, it was shown that post-operative RF closely correlated with a systemic inflammatory response syndrome that attacked the lungs. Respiratory infection readily occurred in patients with RF who underwent long-term tracheal intubation.

In our study, the four patients with postoperative RF remained intubated for at least 8 days, and they all developed respiratory infection. Respiratory infection may aggravate RF, which was confirmed in patients with thoracic aortic aneurysm who underwent TEVAR [28]. However, it remains unclear whether the systemic inflammatory response syndrome and respiratory infection induce a malignant cycle that further induces RF. Fortunately, three of the four patients with postoperative RF recovered. Therefore, the etiology of RF after TEVAR and how to prevent it warrants more study.

## Conclusions

This study shows that malperfusion is the more common indicator for ACBD patients to receive emergency TEVAR. The in-hospital mortality of malperfusion ACBD after TEVAR is closely related to malperfusion and IRI. RF is a common major complication of ACBD after TEVAR and may cause respiratory infection. The correlation between RF and systemic inflammation remain to be further understood.

The most effective prevention and treatment of IRI is to restore blood flow as early as possible. Procedures to improve the perfusion to ischemic end-organs should not be delayed only because of fear of reperfusion injury, unless irreversible ischemia has been proved.

## Abbreviations

ACBD: Acute complicated type B aortic dissection; TEVAR: Thoracic endovascular aortic repair; IRI: Ischemia reperfusion injury;RF: Respiratory failure; CT: Computed tomography; LCCA: Left common carotiod artery; ICU: Intensive care unit

## Competing interests

The authors declare that they have no competing interests.

## Authors’ contributions

JX and WG carried out the conception and design. JX, MZ and WG carried out the analysis and interpretation. JX, MZ, XL, TY, XJ, HZ, YX and LW participated in the data collection. JX participated in the writing the article, the critical revision of the article and the statistical analysis. All authors read and approved the final manuscript.
